# New Delhi Metallo-Beta-Lactamases (NDM)-Carbapenem-Resistant Acinetobacter baumannii Pneumonia: A Case Report

**DOI:** 10.7759/cureus.79198

**Published:** 2025-02-18

**Authors:** Siddartha Guru, Vignesh Harish, Navami Guru, Maimoona Ali, Karoll Cortez

**Affiliations:** 1 Infectious Diseases, Penn State Health Milton S. Hershey Medical Center, Hershey, USA; 2 Critical Care Medicine, Geisinger Medical Center, Danville, USA; 3 Internal Medicine, Greater Baltimore Medical Center, Towson, USA; 4 Internal Medicine, CHI Good Samaritan Hospital, Kearney, USA; 5 Infectious Diseases, Greater Baltimore Medical Center, Towson, USA

**Keywords:** carbapenem-resistant acinetobacter baumannii, clinical case report, new delhi metallo ß lactamases (ndm), pneumonia, resistant gram negative infection

## Abstract

*Acinetobacter baumannii* are Gram-negative aerobic bacteria, that are ubiquitous in the environment. They are difficult to treat given their numerous intrinsic resistance and ability to acquire resistance genes. Infections caused by carbapenem-resistant *A. baumannii* (CR*Ab*) with New Delhi Metallo-beta-lactamases (NDM) are rare in the US but have risen in the past few years. There are limited treatment options available. We present a case of NDM-CR*Ab* pneumonia in a 75-year-old man with a history of penicillin anaphylaxis with severe hypoxia requiring intubation and vasopressor support. In the absence of cefiderocol at the medical facility and the inability to use high-dose ampicillin-sulbactam infusion, a combination of polymyxin B and minocycline was used. Two courses of the combination antibiotic therapy were given, initially four days course then another course of 10 days. After this, he improved clinically and was able to be weaned off the ventilator and vasopressors. However, he experienced acute renal failure from polymyxin B and vancomycin, requiring hemodialysis for four months.

## Introduction

New Delhi Metallo-beta-lactamases (NDM) were first isolated in New Delhi, India, in 2008, in a *Klebsiella pneumoniae* isolate from a urine specimen from a patient with a urinary tract infection [1]. Historically in the US, NDM-producing bacterial infections were rare, the few infections that were reported were mainly from *Escherichia coli* and *Enterobacter* species [2]. The first NDM carbapenem-resistant *Acinetobacter baumannii* (CR*Ab*) case in the US was reported in 2013; with mostly sporadic cases in patients with recent international travel, as only three isolates of NDM-CR*Ab* were reported in 2017 and 2018 [2,3]. However, there have been multiple outbreaks since then, specifically in California, where the California Department of Public Health (CDPH) reported 79 clinical cases of NDM-CR*Ab* between May 2020 and August 2022. All these cases were traced to long-term care facilities, skilled nursing facilities, and hospitals where additional screening led to the identification of 150 more NDM-CR*Ab*-positive isolates [4]. Current Infectious Disease Society of America (IDSA) 2024 guidelines for treating antimicrobial-resistant Gram-negative infections recommend using sulbactam-durlobactam in combination with a carbapenem for CR*Ab *infections [5]. This combination is effective against class A, C, and D beta-lactamases, which include OXA-23, OXA-40, and OXA-58 but not against class B beta-lactamases such as NDM. IDSA recommends using ceftazidime-avibactam with aztreonam for the treatment of NDM infections, which works with *Enterobacterales *but not *A. baumannii* and is intrinsically resistant to aztreonam. Currently, per antibiotic-resistance laboratory network (ARLN) data, NDM-CR*Ab* only accounts for about 2.7% of all CR*Ab* cases in the US in 2023 but that equates to 202 cases compared to only three cases in 2018 [2]. There are no current guidelines for the treatment of NDM-CR*Ab* infections. Also, limited published literature regarding the management and outcomes of NDM-CR*Ab* infections. In this case report, we share our experience in managing NDM-CR*Ab* pneumonia in a patient with no history of international travel.

## Case presentation

**Figure 1:**
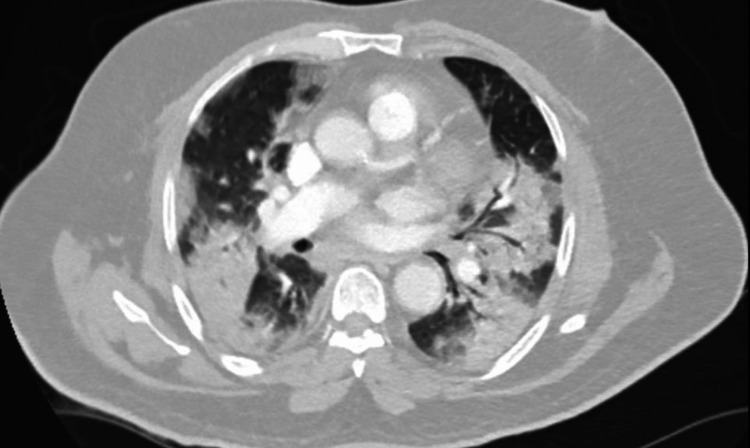
CT chest angiogram with contrast CT chest shows ground glass and consolidative opacities bilaterally with trace pleural fluid present bilaterally.

A 75-year-old man with a history of hypertension, coronary artery disease status post stent placement, and penicillin allergy presented to the emergency department (ED) with six days of sore throat, dry cough, progressive shortness of breath, and two days of fevers and chills. On admission, he was hypertensive, tachycardic, tachypneic, and hypoxic on room air (Table 1). During the physical exam, decreased breathing sounds were noted at bilateral lung bases. Labs showed no leukocytosis, elevated creatinine, HgbA1c, CRP, and procalcitonin (Table 2). Computed tomography (CT) chest angiogram revealed ground glass and consolidative opacities in bilateral lung fields consistent with COVID-19 pneumonia (Figure 1). The respiratory pathogen panel was positive for SARS-CoV-2.

**Table 1:** Labs on admission

Labs	Value	Reference ranges
White blood cell count	7.52 x 10^3^/µL	4.00 - 11.00 x 10^3^/µL
Creatinine	1.57 mg/dL	0.50 - 1.20 mg/dL
HgbA1c	7.3%	< 5.7%
CRP	23.07 mg/dL	≤ 0.50 mg/dL
Procalcitonin	0.59 ng/mL	≤ 0.10 ng/mL

**Table 2:** Vitals on admission

Vitals	Value
Temperature	37.1º C
Blood pressure	187/86
Heart rate	125 beats per minute
Respiratory rate	36 breaths per minute
Oxygen saturation	74% on room air

He was started on dexamethasone for COVID-19 pneumonia and ceftriaxone and azithromycin was added on empirically for possible superimposed bacterial pneumonia. Blood cultures were drawn prior to antibiotics but no sputum cultures obtained at this time given that it was a non-productive cough. His oxygen saturation continued to slowly fall, with increased work of breathing, thus he was intubated on hospital day 4 (HD).

On HD 5, sputum cultures were obtained due to increased secretions from the endotracheal tube. His oxygen requirements on the ventilator decreased over the next 48 hours and he was extubated to 6 L nasal canula on HD 7. Repeat sputum cultures were obtained on HD 7 for worsening productive cough.

On HD 8, sputum cultures from HD 5 showed light growth of *A. baumannii* with susceptibilities as seen in Table 3. Carbapenemase real-time PCR panel detected NDM gene. Extended susceptibilities to the NDM-CR*Ab* were requested by the infectious disease team including minocycline, colistin, tigecycline and polymyxin B via disk diffusion method (Table 4). Sputum cultures from HD 7 showed heavy growth of *A. baumannii*. He had no fever or leukocytosis, but his oxygen requirement had increased over the last 24 hours, and he had productive cough now compared to dry cough on admission. We were concerned about a new developing pneumonia; thus, a decision was made to treat.

**Table 3:** Acinetobacter baumannii sputum culture sensitivities MIC: Minimal inhibitory concentration, TMP-SMX: Trimethoprim/sulfamethoxazole

	Acinetobacter baumannii	
Antibiotics	MIC	Interpretation
Amikacin	> 32 mcg/mL	Resistant
Ampicillin/sulbactam	> 16/8 mcg/mL	Resistant
Cefepime	> 16 mcg/mL	Resistant
Ceftazidime	> 16 mcg/mL	Resistant
Ceftriaxone	> 32 mcg/mL	Resistant
Ciprofloxacin	> 2 mcg/mL	Resistant
Gentamicin	> 8 mcg/mL	Resistant
Levofloxacin	> 4 mcg/mL	Resistant
Meropenem	> 8 mcg/ml	Resistant
Tobramycin	> 8 mcg/mL	Resistant
TMP-SMX	> 2/38 mcg/mL	Resistant

**Table 4:** Extended susceptibilities for Acinetobacter baumannii with disk diffusion NA: Not applicable (there are no CLSI breakpoints available for the sensitivity of *A. baumannii* for Colistin and Polymyxin B).

	Disk diffusion	
Colistin	0 mm	NA
Minocycline	18 mm	Susceptible
Polymyxin B	11 mm	NA

Since there were no guidelines for the treatment of NDM-CR*Ab* infections, the Infectious Disease Society of America (IDSA) guidelines for other (non-NDM) CR*Ab* infections recommended the combination of at least two antibiotics with known invitro activity including high dose ampicillin-sulbactam but our patient had history anaphylaxis reaction to penicillin. The other recommendation was to use polymyxin B in combination with at least one other agent. He was started on intravenous minocycline 200 mg loading dose followed by 100 mg every 12 hours and intravenous polymyxin B 2.12 million units loading dose followed by 1.5 million units every 12 hours.

On HD 9, he was reintubated for worsening hypoxia and was hypotensive requiring vasopressors. However, on this antibiotic combination, his oxygenation improved and was weaned off vasopressors. His renal function worsened; hence, the antibiotics were discontinued on HD 11.

On HD 14, developed new fever with increasing leukocytosis hence work up with blood and urine cultures were obtained followed by sputum cultures on HD 15. The sputum cultures grew heavy growth of NDM-CR*Ab* but the patient was requiring minimal oxygen support on ventilator and no worsening infiltrates on chest x-ray; so, antibiotics for NDM-CR*Ab* were not restarted. Blood and urine cultures remained negative, leukocytosis and fevers resolved after 48 hours. On HD 24, he was extubated to high flow nasal canula.

He became febrile on HD 27, started on empiric intravenous meropenem 500 mg every six hours. With extensive workup, he was found to have acute acalculous cholecystitis. His respiratory symptoms worsened with imaging showing worsening bilateral infiltrates; hence, sputum cultures were sent, which grew moderate MRSA and moderate NDM-CR*Ab* on HD 32. Vancomycin was initiated; however, NDM-CRAb was not treated, as it was likely to be colonization at the time. On HD 34, he underwent tracheostomy and PEG tube placement. On HD 36, he underwent IR-guided cholecystectomy tube placement (Table 5).

**Table 5:** Summary of hospital events HD: Hospital stay

HD	Important events
1	Dexamethasone for COVID pneumonia and Ceftriaxone and azithromycin for superimposed bacterial pneumonia. Blood cultures #1 obtained
4	Intubated and placed on ventilator mode PRVC
5	Sputum culture #1 obtained for increased endotracheal secretions. Blood cultures #1 – no growth
7	Extubated to 6 L nasal canula and sputum culture #2 for worsening productive cough
8	Sputum culture #1 – grew light growth of Acinetobacter baumannii Sputum culture #2 – grew heavy growth of Acinetobacter baumannii Polymyxin B and minocycline started
9	Intubated for worsening hypoxia with increased pulmonary infiltrates on imaging and vasopressors started
10	Weaned off vasopressors
11	Decreased oxygen requirements. Minocycline & polymyxin B stopped due to worsening renal function. Nasopharyngeal and oropharyngeal bleeding treated with tranexamic packing and Afrin spray.
12	Endoscopy showed Dieulafoy lesion in stomach treated with epinephrin injection and hemostatic clip.
14	New fever – blood cultures #2 obtained
15	Sputum culture #3 for the continued fever
16	Sputum culture #3 – grew heavy growth of Acinetobacter baumannii
18	Blood cultures #2 – no growth
24	Extubated to high flow nasal canula
27	New fever, meropenem started
29	Abdominal ultrasound and HIDA scan showed findings consistent with acalculous cholecystitis
30	Sputum cultures #4 obtained for worsening respiratory symptoms
32	Sputum culture #4 – grew moderate growth of Acinetobacter baumannii and MRSA. Vancomycin started
34	Tracheostomy and PEG tube placement
36	IR guided cholecystostomy tube placement
38	Worsening hypoxia and requiring vasopressors despite being on meropenem and vancomycin. Started on polymyxin B and minocycline
40	Vasopressors stopped
48	Polymyxin and minocycline stopped and weaned to trach collar
69	Discharged to subacute rehab facility

**Figure 2:**
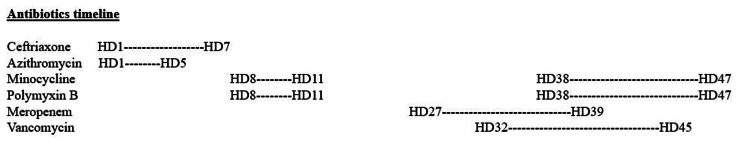
Antibiotics timeline

Over the next 48 hours, his oxygen requirements on ventilator continued to increase despite being on meropenem and vancomycin. In addition, his procalcitonin was elevated at 4.06 ng/mL, so he was started on minocycline and polymyxin B on HD 38. On this regimen was continued for 10 days (Figure 2), his oxygen requirement on the ventilator improved transitioning to trach collar and was weaned off vasopressors. His procalcitonin improved to 1.33 ng/mL. Unfortunately, his renal function worsened throughout the hospitalization, likely multifactorial in nature, hypotension from gastrointestinal bleed, and antibiotics specifically vancomycin and polymyxin B. He required continuous renal replacement therapy (CRRT) and then transitioned to hemodialysis for the next four months before his renal function recovered.

## Discussion

*A. baumannii* is a Gram-negative nonfermenting aerobic coccobacilli, ubiquitous in the environment, typically found in soil and water animals and colonized in humans [6]. The pathogenicity of *A. baumannii* is based on its many virulence factors and resistance mechanisms, including its ability to form biofilms and produce a large variety of beta-lactamases, some of which are intrinsic, and others acquired [7]. Biofilms allow it to adhere to surfaces of foreign material, leading to catheter-associated UTI, intravascular device-associated bloodstream infection, and, more commonly, ventilator-associated pneumonia [6]. The SENTRY antimicrobial surveillance program reviewed the susceptibility of 1029 *A. baumannii* isolates between 2014 and 2021 with sensitivities as follows: 86.2% to minocycline, 59.3% to levofloxacin, and 61.5% to meropenem [8].

NDM is a class B beta-lactamase, which hydrolyzes most beta-lactams, including penicillin, cephalosporins, and carbapenems but not aztreonam [9]. NDM-producing Gram-negative organisms have more than one type of beta-lactamases, such as extended-spectrum beta-lactamases (ESBLs) in addition, which would break down aztreonam. To counteract this, the current IDSA guidelines recommend the combination of ceftazidime-avibactam to inhibit ESBLs to protect aztreonam which would then be free to act on the Gram-negative organisms and not be broken down by NDM [5]. Unfortunately,* A. baumannii *is intrinsically resistant to aztreonam due to the production of AmpC-type beta-lactamases; thus, the combination of ceftazidime-avibactam and aztreonam would not work. The current IDSA recommendation for CR*Ab* states to use sulbactam-durlobactam in combination with meropenem. The durlobactam would inhibit the KPCs and OXA-48 beta-lactamases so meropenem and sulbactam could work on the *Acinetobacter* but unlike KPCs and OXA-48, NDM is not inhibited by durlobactam, thus, this combination would not work either. The old IDSA recommendation mentioned in the case presentation, the combination of high-dose ampicillin-sulbactam and another active agent is now a second-line regimen recommendation that could not be used in our patient, due to his anaphylaxis allergy to penicillin.

In a retrospective analysis of CR*Ab* infection in India, 23/50 patients had NDM-CR*Ab*, who were treated with either polymyxin monotherapy or polymyxin combination therapy with one of the following: meropenem, minocycline, sulbactam, amikacin, or ciprofloxacin [10]. The overall mortality rate was about 60% which is consistent with another study by Singh et al., which noted a mortality rate of 75% [11]. A small case series involving 13 patients with NDM-CR*Ab* infections treated with only cefiderocol or cefiderocol with colistin combination. One of the two patients with monotherapy died compared to one out of 10 patients on the combination [12]. This does look like a promising combination regimen: but it is unclear if we can extrapolate to the larger patient population. The results of the CREDIBLE-CR trial showed that patients with Gram-negative infection treated with cefiderocol have a higher mortality rate of 34% compared to 18% on the best available therapy [13].

Our patient was the third case of NDM-CR*Ab* ever isolated from our hospital. There was an outbreak of NDM-CR*Ab* from a specific skilled nursing facility in our state; seven cases of NDM-CR*Ab* admitted to our hospital were traced back to that skilled nursing facility. Our patient, however, came from home and to our knowledge had no direct contact with that skilled nursing facility. The first patient with NDM-CR*Ab* infection from the skilled nursing facility arrived nine days prior to our patients to the hospital. He was in the ICU for septic shock with the urinary source of infection and despite antibiotics succumbed to the infection on the day before our patient arrived at the hospital in ED. According to the infection control investigation, the two occurrences were unrelated because the patients were in different ICU rooms.

From our experience with this patient, on the combination of polymyxin B and minocycline, the was weaned off the ventilator and vasopressors. There is a risk of nephrotoxicity associated with this regimen, more so in patients with higher BMI and on vasopressors as was seen in our patient, where polymyxin B has an increased risk of causing renal impairment. The patient required hemodialysis for four months before his renal function returned to baseline. Given the rise in NDM-CR*Ab* infections in the US, further studies are required to investigate possible antibiotic combination regimens or new antibiotics for the treatment of NDM-CR*Ab*.

## Conclusions

Carbapenemases, like NDMs, are more commonly found in carbapenem-resistant *Enterobacterales* but rarely in CR*Ab*. NDM-CR*Ab* could have all four classes of beta-lactamases along with other resistance mechanisms, including efflux pumps and target site modification from gene mutations, making infections clinically challenging. There are limited options for the treatment of NDM-CR*Ab*; only cefiderocol is effective against NDM-CR*Ab*. In community hospitals, which do not have access to cefiderocol or patients who are unable to tolerate the medication, treatment becomes challenging. We successfully treated our patient’s NDM-CR*Ab* pneumonia with a combination of polymyxin B and a minocycline regimen. Though it was an effective regimen, and the patient improved clinically, it was nephrotoxic. In the absence of an alternative, the combination of polymyxin B and minocycline could be used for NDM-CR*Ab* pneumonia.

## References

[1] Yong D, Toleman MA, Giske CG, Cho HS, Sundman K, Lee K, Walsh TR (2009). Characterization of a new metallo-beta-lactamase gene, bla(NDM-1), and a novel erythromycin esterase gene carried on a unique genetic structure in Klebsiella pneumoniae sequence type 14 from India. Antimicrob Agents Chemother.

[2] (2025). ARLN explorer. https://arpsp.cdc.gov/arln-explorer.

[3] Medrzycki M, Stanton RA, Rankin DA (2023). Molecular epidemiology of NDM-producing Acinetobacter baumannii in the US—October 2013—March 2022. Open Forum Infect Dis.

[4] (2025). LHD IP forum course highlights: NDM-CRAb outbreak in California. https://www.cdph.ca.gov/Programs/CHCQ/HAI/CDPH%20Document%20Library/LHD_IP_ForumCourseHighlights_NDM-CRAB_OutbreakInCA_051823.pdf.

[5] Tamma PD, Heil EL, Justo JA, Mathers AJ, Satlin MJ, Bonomo RA (2024). Infectious Diseases Society of America 2024 guidance on the treatment of antimicrobial-resistant Gram-negative infections. Clin Infect Dis.

[6] Hochman S, Phillips M (2020). Acinetobacter species. Mandell, Douglas, and Bennett's Principles and Practice of Infectious Diseases. 222nd ed..

[7] Lee CR, Lee JH, Park M (2017). Biology of Acinetobacter baumannii: pathogenesis, antibiotic resistance mechanisms, and prospective treatment options. Front Cell Infect Microbiol.

[8] Pfaller MA, Shortridge D, Carvalhaes CG, Castanheira M (2023). Trends in the susceptibility of U.S. Acinetobacter baumannii-calcoaceticus species complex and Stenotrophomonas maltophilia isolates to minocycline, 2014-2021. Microbiol Spectr.

[9] Dortet L, Poirel L, Nordmann P (2014). Worldwide dissemination of the NDM-type carbapenemases in Gram-negative bacteria. Biomed Res Int.

[10] Prayag PS, Patwardhan SA, Joshi RS, Panchakshari SP, Rane T, Prayag AP (2023). Enzyme patterns and factors associated with mortality among patients with carbapenem resistant Acinetobacterbaumannii (crab) bacteremia: real world evidence from a tertiary center in India. Indian J Crit Care Med.

[11] Singh S, Verma A, Venkatesh V, Verma S, Reddy DH, Agrawal A (2024). The clinical impression of NDM-producing Acinetobacter baumannii in intensive care units of the University Referral Hospital in North India. Indian J Crit Care Med.

[12] Travi G, Peracchi F, Merli M (2024). Cefiderocol-based regimen for Acinetobacter NDM-1 outbreak. Antibiotics (Basel).

[13] Bassetti M, Echols R, Matsunaga Y (2021). Efficacy and safety of cefiderocol or best available therapy for the treatment of serious infections caused by carbapenem-resistant Gram-negative bacteria (CREDIBLE-CR): a randomised, open-label, multicentre, pathogen-focused, descriptive, phase 3 trial. Lancet.

